# Research and Development of a 3D-Printed Dynamic Finger Flexion Orthosis for Finger Extension Stiffness—A Preliminary Study

**DOI:** 10.3390/bioengineering11040339

**Published:** 2024-03-29

**Authors:** June-Tzu Yu, Yi-Chao Huang, Chen-Sheng Chen

**Affiliations:** 1 Department of Physical Therapy and Assistive Technology, National Yang Ming Chiao Tung University, Taipei 112, Taiwan; girlgirl630@gmail.com; 2 Department of Orthopaedics and Traumatology, Taipei Veterans General Hospital, Taipei 112, Taiwan; 3 Department of Orthopaedics, School of Medicine, National Yang Ming Chiao Tung University, Taipei 112, Taiwan

**Keywords:** finger extension stiffness, post-operative rehabilitation, additive manufacturing, dynamic hand orthosis, hand functional activity

## Abstract

Finger extension stiffness is a common post-traumatic complication that results in the hand’s functional impairment. In clinical practice, a dynamic splint enables the patient to stretch the affected finger independently. However, current dynamic splints have drawbacks, such as limited stretching efficacy, and interfere with the hand’s functional activities. Therefore, this study aimed to develop a dynamic finger flexion orthosis capable of stretching each finger joint using additive manufacturing (AM) technology, thereby enabling hand functional activity, and analyze the clinical improvement in the range of motion (ROM). One subject with a hand fracture was recruited while undergoing a 7-week home-based rehabilitation program for the orthosis. The outcome measurements included the total active motion (TAM), the tip-to-finger distance (TPD), and the score on the Disability of Arm, Shoulder, and Hand (DASH) questionnaire. The results show that the TAM of the participant’s fingers increased by 72.7 degrees on average, the TPD decreased by 3.5 cm on average, and the DASH score decreased to 9.5 points. The 7-week home-based rehabilitation program for the orthosis resulted in a 53.6% increase in the TAM on average. The developed orthosis improved hand function and enabled a more complete ROM in finger flexion.

## 1. Background

Finger extension stiffness is one of the most common complications of hand injuries; its incidence ranges from 13% to 41% [1,2,3,4] among different types of hand surgeries. Patients with finger extension stiffness are likely to suffer from a loss of range of motion (ROM) of the finger joints, which makes finger extension difficult, followed by a sequential disability in hand activities [5]. The main cause of this post-traumatic condition is adhesion and scar tissue formation during the wound-healing process. This occurs mostly in the proliferative phase when the patients are in a necessary post-operative immobilization phase. Collagen deposition occurs on the injured site and the adjacent tissue, resulting in a stiffened area in the hand. In clinical rehabilitation programs, interventions used in the hospital setting to eliminate tissue stiffness include modalities, continuous passive motion machines (CPMs), and manual joint mobilizations. Home-based rehabilitation programs consist of exercises and orthosis use [6,7].

Among these treatments, the use of orthosis plays an important role in assisting patients to maximize the effects of rehabilitation. Previous studies have shown that rehabilitation programs with an orthosis, such as a dynamic splint, can effectively improve the ROM of the finger joints. The patient can use the outer force that stretches the stiff tissue to increase the span and extension of the stiff tissue on their own [8,9].

Additive manufacturing (AM), also known as three-dimensional printing (3DP), technology has been widely adopted in the medical field in recent decades to meet the demands of individual differences [10,11,12] and has been featured in numerous studies on hand orthosis.

Regarding the concept of printing a static progressive orthosis for hand rehabilitation, Huang et al. designed two different devices for two patients: one with a distal radius fracture and another after surgery for flexor pollicis longus (FPL) tenolysis. The ROM of the wrist extension of the former subject and the ROM of the MCP and IP joints of the latter subject were improved after 2 months of follow-up [13]. To address the inconvenience of traditional plaster for a patient after an upper limb fracture, Chen et al. proposed a 3DP splint customized to each subject as a solution and found that a 3DP static splint provided a more comfortable experience and better ROM of the injured joint than traditional plaster in the application of postoperative immobilization [14]. Three-dimensional printing technology is one component of the manufacturing of novel myoelectric hand orthoses for patients with spinal cord injury (SCI) that allow them to handle objects, and it is used to generate sophisticated parts of exoskeletons for other devices. One study showed that a myoelectric hand orthosis could restore hand function and ADL in people with SCI [15]. Chen et al. developed a passive 3D-printed device to help stroke patients practice gripping. They recruited six participants for home rehabilitation training and measured changes in grip strength and function. The results showed improvements in all the measured outcomes [16]. The research on hand orthosis mentioned above proved that AM technology enabled the manufacture of concrete structures with enough hardness to fulfill highly customized and delicate design concepts. Moreover, the clinical outcomes of 3DP hand devices were comparable or superior to those of traditional solutions [16].

Despite their promised therapeutic effects, current dynamic splints for finger extension stiffness, in either clinical practice or academic studies, are not uniform among different institutions and yet share the same drawbacks, such as interfering with the hand’s functional activity, causing incomplete flexion stretching movement of the finger joints, and complicated wearing steps as a temporary measure for patients. Therefore, this study aimed to develop a novel dynamic finger flexion orthosis to address these drawbacks and investigate its therapeutic effect on patients with finger extension stiffness.

## 2. Methods

To develop the new device and test its feasibility, the following are descriptions of the mechanism and processing of the device, the rehabilitation program, and the outcome measurement parameters.

### 2.1. The Design of the 3D-Printed Finger Flexion Dynamic Orthosis

**Figure 1:**
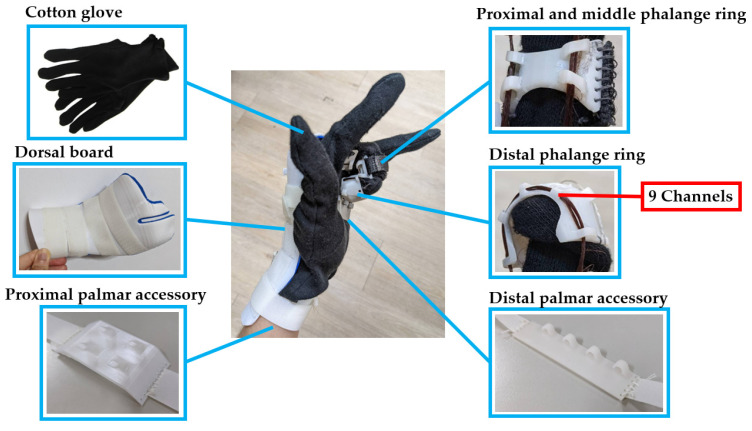
Components of the dynamic finger flexion orthosis.

The developed orthosis comprised a glove with phalange rings, 2 sets of elastic lines, a dorsal board, and 2 accessories on the palmar side (Figure 1).

Unlike traditional dynamic splints, the device adapted the concept of ‘putting on a glove’ for a simpler wearing process. To properly apply the tension force of the elastic line to the joint center, the device contained three 3DP phalange rings in each target finger as a pulley system. The elastic line ran through short channels mounted on the phalange rings. Nine channels were mounted on the distal phalange ring, five on the palmar side, and four on the dorsal side. Four channels were mounted on the palmar side of the middle and proximal phalange rings. Elastic Velcro was sewn onto the phalange rings for tightness adjustment. All the phalange rings were sewn onto a cotton glove.

**Figure 2:**
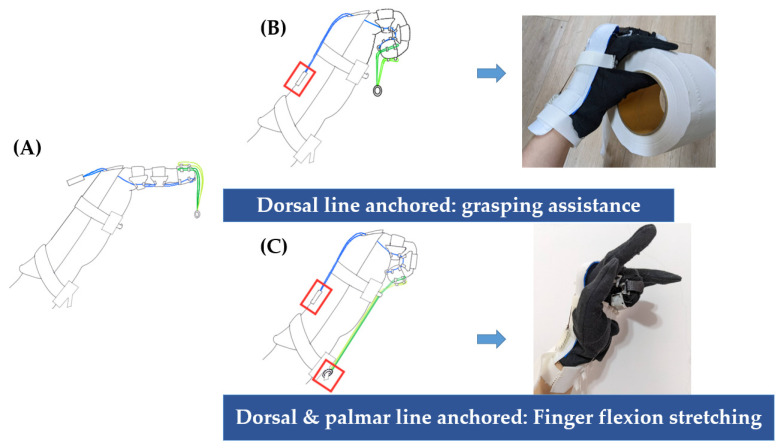
Configurations of the dorsal and palmar elastic lines. (**A**) Starting state with no elastic line tightened; (**B**) tightening the dorsal elastic line (blue line) and anchoring it on the dorsal board (red hollow square), practicing functional grasping movement; and (**C**) tightening both the dorsal elastic line (blue line) and palmar elastic line (green line), performing stretching exercise for stiff joints.

This device contained 2 sets of elastic lines designed for finger flexion movement in a specific usage context. The dorsal line ran from the palmar side of the fingertip to the dorsal side of the hand. The tightened dorsal line was anchored onto the dorsal board by attaching the Velcro piece connected to its end. Two palmar lines were hung from the dorsal channels of the distal phalange ring and knotted together at the end with a metal ring. Tightening the dorsal line alone exerted a traction force that facilitated finger flexion. Unlike with a traditional dynamic splint, the user was allowed to practice grasping objects while wearing the device on their hand without any lines on the palm. Both the dorsal and palmar lines were tightened during the device’s second usage. The tightened palmar line was pulled to reach the palmar side of the forearm; thus, the traction force was augmented for the stretching exercise (Figure 2).

**Figure 3:**
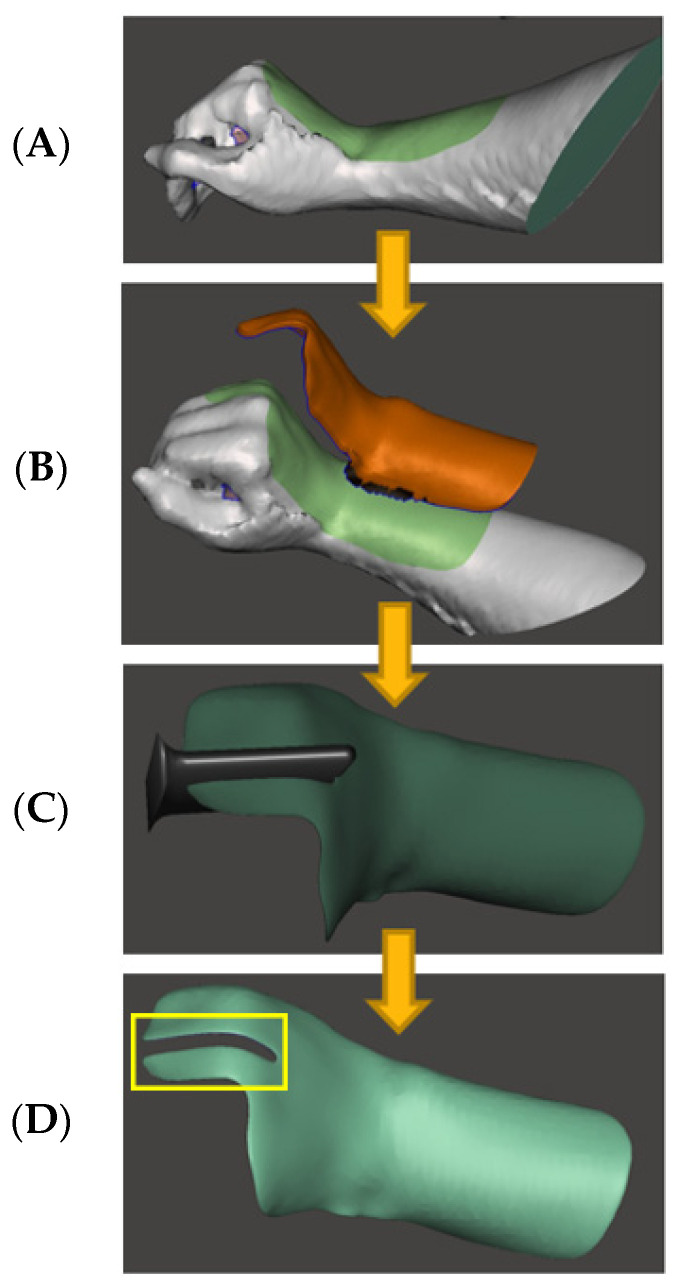
Process of generating a customized dorsal board: (**A**) import the hand model from the iPad scanner, (**B**) extract the shape of the hand using Meshmixer 3.5 software, (**C**) remove redundant volume to create a slot to allow cable sliding, and (**D**) smooth the dorsal board. (The yellow box indicate the location of a slot).

The 3DP dorsal board was obtained by scanning the participant’s hand using an iPad (Ipad5, Apple Co., Cupertino, CA, USA) and a 3D scanner (Model SA15, Occipital Co., Cupertino, CA, USA), followed by the computer-aided processing of the 3D model (Figure 3). The 3DP dorsal board included two main functions: (1) stabilizing the glove in case it slipped out during the exercise, and (2) serving as a platform for the dorsal elastic line and palmar accessories to settle by attaching a Velcro band.

The edges of the distal and proximal palmar accessories were connected to a Velcro band. Four channels with gaps were mounted on the distal palmar accessory to fix the palmar elastic line close to the palm and ensure that the finger joints were stretched to the maximum. Four hooks were mounted on the proximal palmar accessory to hold the metal ring knotted at the end of the palmar elastic line.

### 2.2. Fabrication of the 3D-Printed Parts of the Finger Flexion Dynamic Orthosis

#### 2.2.1. Dorsal Board

The dorsal board was produced using a fused deposition modeling (FDM) printer (CR-200B, Creality Co., Shenzhen, China) with polylactic acid (PLA) material. The dorsal board was attached to bony prominences on the back of the hand, such as the metacarpophalangeal joints, the radiocarpal joint, and the ulnocarpal joint. Here, 12 mm of foam padding was added as a cushion. The dorsal board was printed at 110% of the original model size to provide the space needed to attach the foam padding and thickness of the cotton glove for the user.

#### 2.2.2. Phalange Rings and Distal Palmar Accessory

The phalange ring and distal palmar accessory were 3D-printed using a high-precision stereolithography apparatus (SLA) printer (Sonic Mighty 8k, Phrozen Co., Hsinchu, Taiwan) made of highly resilient UV-curable resin (BASF, Phrozen Co., Hsinchu, Taiwan) composed of 4-acryloyl morpholine (4-AM) and polymeric urethane acrylate (PUA) to withstand the deformation caused by the tension of the elastic Velcro when worn. The specific parameter settings for this resin were as follows: the bottom layer count was 6, the bottom layer exposure time was 35 s, the layer height was set to 0.05 mm, and the normal layer exposure time was 10.5 s. The phalange rings were printed at 115% of the original model size to accommodate the increased finger circumference while wearing the cotton glove. The inner diameter of the original model of phalange rings was equal to the largest width of each phalange.

#### 2.2.3. Proximal Palmar Accessory

The proximal palmar accessory was printed using an FDM printer (CR-10 V3, Creality Co., Shenzhen, China). Thermoplastic polyurethane (TPU) material was chosen due to its flexibility to avoid causing discomfort when tightening the wrist. The sizes of the distal and proximal palmar accessories were tested in advance to be universal for different hand and forearm sizes.

### 2.3. Participant

This pilot study was approved by the Institutional Review Board of Taipei Veterans General Hospital (plan number: TPEVGH2021-07-023AC). The participant provided informed consent before the beginning of the program. The inclusion criteria set for this case study included the patient (1) having post-operative finger extension stiffness caused by a traumatic hand injury and (2) being within 3 months of the operation. The exclusion criteria included the patient (1) having an untreated hand injury or revision surgery and (2) having finger extension stiffness caused by a specific disease, such as arthritis, trigger finger, Dupuytren’s disease, or secondary to another pathology, such as diabetes mellitus. Based on these criteria, one patient was recruited for this pilot study. The patient’s injured finger was unconfirmed.

### 2.4. Home-Based Rehabilitation Program

The home-based rehabilitation program lasted for 7 weeks, corresponding to the proliferation phase of the wound-healing process. The participant was asked to perform the following exercises: (1) tightening the dorsal elastic line only and practicing a dynamic grasping movement (with or without an object) for 150 repetitions per day, and (2) tightening both the dorsal and palmar elastic lines, keeping all finger joints stretched in the maximal flexion position for at least 5–10 min twice a day. The participant was encouraged to perform more than the minimum requirement of rehabilitation exercises.

**Figure 4:**
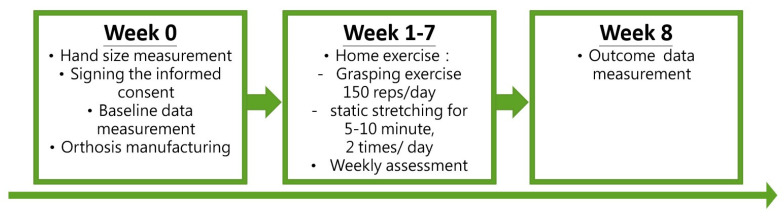
Rehabilitation program.

The outline of the rehabilitation program and parameter measurement is depicted in Figure 4.

### 2.5. Biomechanical Evaluation

**Figure 5:**
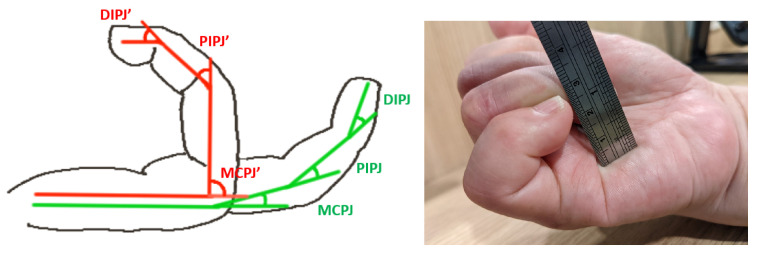
Measurement of TAM and TPD.

This study aimed to present the proposed orthosis and explore its clinical improvement in the ROM of the finger joints by measuring the total active motion (TAM), which represented the sum of the active flexion ROM of the metacarpophalangeal (MCP) joint, proximal interphalangeal (PIP) joint, and distal interphalangeal (DIP) joint, as shown in Figure 5. The measurement was performed using a goniometer. The calculation of the TAM is defined as follows:

TAM = (MCPJ′ − MCPJ) + (PIPJ′ − PIPJ) + (DIPJ′ − DIPJ)

Note: MCPJ, PIPJ, and DIPJ are defined during flexion in the resting position (Figure 5, green line). MCPJ′, PIPJ′, and DIPJ′ are defined during the maximum flexion at the greatest force (Figure 5, red line).

The tip-to-palm distance (TPD) parameter was also used to quantify hand function and was obtained using a meter to measure the linear distance between the fingertip and palm. The first measurements of the TAM and TPD of each injured finger were taken as baseline data three weeks after the surgery (Week 0).

The subjective functional outcome was obtained by using the Taiwan version of the Disabilities of the Arm, Shoulder, and Hand questionnaire (DASH), in which the activity, symptom, and work sections were tested on a 30-item scale about the patient’s health status [17]. These items were used to evaluate different physical activities consisting of arm, shoulder, or hand problems (21 items); the severity of the symptoms of pain or activity-related pain (5 items); and the effects on social activities, work, sleep, and self-image (4 items). Each item was given a score ranging from zero (no disability) to five (the most severe disability). These items were summarized as a DASH score. A lower DASH score represented a better health status.

## 3. Results

The results of this study contained information about the subject and two main outcome measurements: the ROM of the finger joints, representing the therapeutic effect of the stretching function provided by the device, and the DASH score to show if the grasping exercise allowed by the device generated actual improvement in the hand’s daily functional activity. The TPD was used as an intuitive and parallel parameter to support both of these outcome measurements.

This study recruited a 59-year-old woman with injuries and stiffening in the three fingers of her right hand, middle finger, ring finger, and little finger. The demographic information is recorded in Table 1.

### 3.1. TAM and ROM

**Figure 6:**
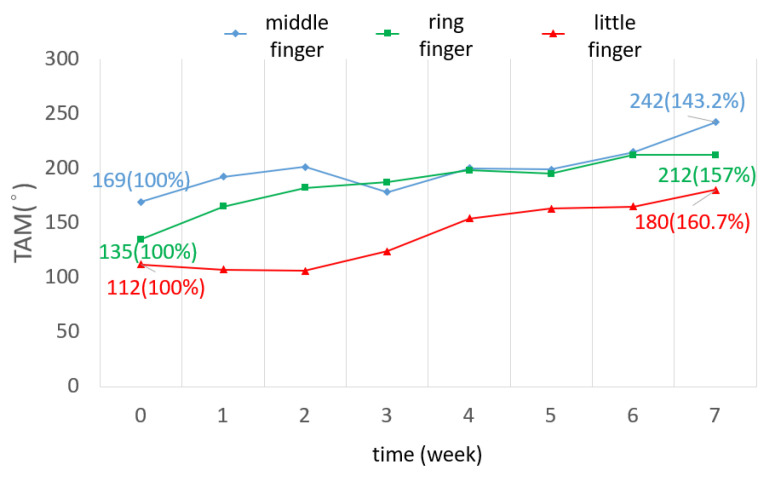
TAM of three fingers over 7 weeks.

**Figure 7:**
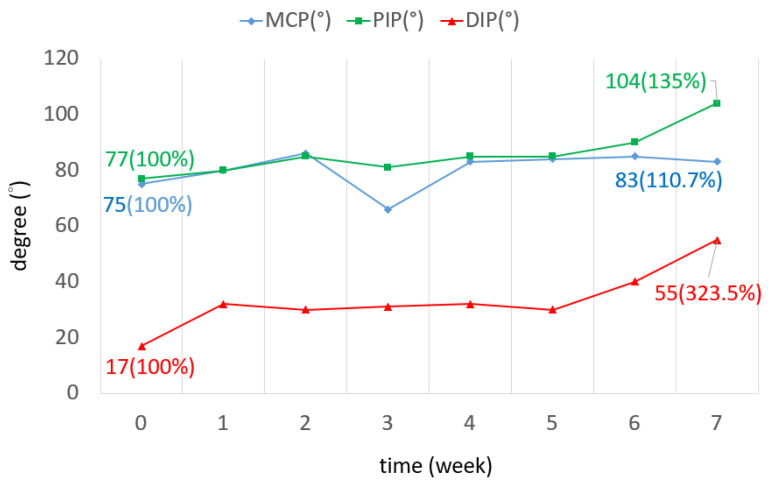
TAM of the middle finger over 7 weeks.

**Figure 8:**
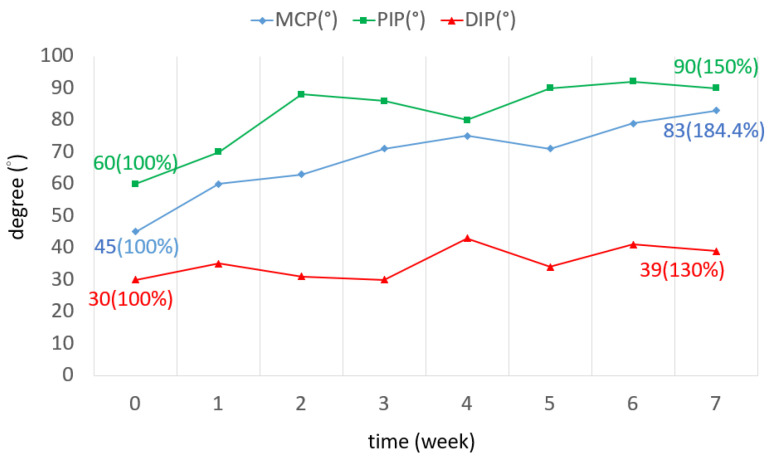
TAM of the ring finger over 7 weeks.

**Figure 9:**
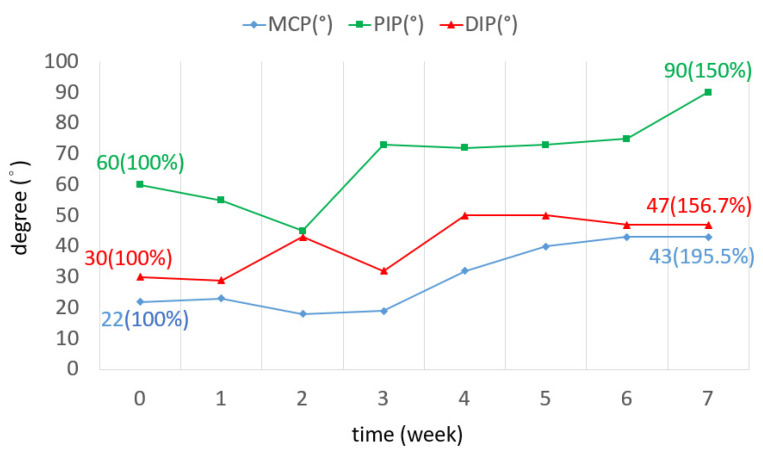
TAM of the little finger over 7 weeks.

The TAM of all three fingers was increased by 72.7 degrees on average. The ROM increased by 60% in the little finger, as shown in Figure 6. The ROM of all joints in the three fingers was improved. In the middle finger, the ROM of the DIP joint in the seventh week was three times greater than that of the DIP joint in week 0. The ROM of the PIP joint was increased by 28 degrees (Figure 7). The ROM of the MCP joint in the ring finger was 38 degrees more than the original (Figure 8). The ROM of the PIP joint in the little finger was increased by 30 degrees (Figure 9). The ROM of most joints in the three fingers improved after the third week.

### 3.2. TPD

**Figure 10:**
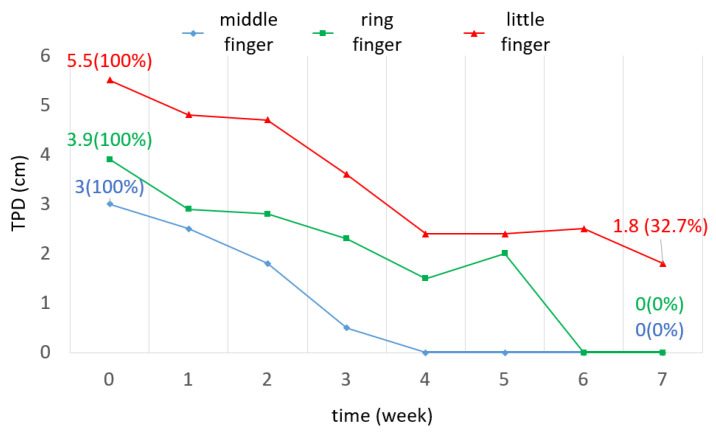
TPD of three fingers over 7 weeks.

The TPD of the middle and ring fingers decreased to 0 cm, while the TPD of the little finger decreased to 1.8 cm (Figure 10). This indicated that the middle and ring fingers recovered to normal after wearing the orthosis.

### 3.3. DASH Score

A lower DASH score represented a better upper limb functional outcome. In this study, the DASH score was reduced to 9.5 points, a 16.4 decrease. The scores of the I, II, and III sections were reduced to 10.2, 7.1, and 0 points, respectively (Table 2). This indicated that a remarkable improvement in the upper limb functional outcome was obtained.

## 4. Discussion

This study aimed to develop a dynamic finger flexion orthosis that could stretch each finger joint, allow hand functional activity to be performed using AM technology, and analyze its clinical improvement in the ROM and subjective functional outcomes.

### 4.1. TAM

After 7 weeks of rehabilitation, the TAM of the three fingers was 46.3 degrees more than the functional ROM [18] on average; however, it was 48.7 degrees less than the normal ROM of finger flexion advised by the American Medical Association in the fifth edition of their guidelines.

The increase in TAM obtained in this study was 51.7 degrees more than that in a previous study that used a dynamic splint to treat finger extension stiffness [19,20]. An explanation for this inconsistency might be the differences between the devices. The dynamic splints used in Glasgow et al.’s and Jun et al.’s studies had a limited stretching effect, which was applied only to the MCPJ, while the device proposed in this study was designed to apply traction force to each finger joint. Moreover, the outcome measurements were performed in the 6th and 12th weeks of Jun et al.’s experimental procedure. The increase in the TAM between these two time points may have been insignificant because the rehabilitation began in the third week.

Release surgery is an alternative to conventional treatments for joint stiffness. A previous study [21] on the TAM follow-up outcome of this operation found a 41-degree difference in the TAM, which was 31.7 degrees less than our finding. This suggests that the patient with finger extension stiffness of similar severity to that of the participant in this study may achieve better TAM progress by using the proposed device instead of undergoing a risky invasive procedure.

#### 4.1.1. MCP

After 7 weeks, the average flexion ROM of the MCP joint in the three fingers exceeded the functional angle [18] by 7.7 degrees, except for the little finger, which fell short by 19 degrees. The average ROM of the three fingers was 20.3 degrees less than the normal joint angle, with the little finger showing the greatest deficit of 47 degrees.

In previous studies on conventional treatments [13,20,22], the average pre–post-intervention difference was 14.2 degrees. Meanwhile, in this study, the average pre–post-difference was 22.3 degrees, surpassing that obtained in previous research. However, the MCP joint ROM in the middle finger only improved by 8 degrees, which was 6.2 degrees less than in the prior literature. This limited improvement might be due to the ceiling effect caused by minimal initial damage to the middle finger.

The designs of the dynamic splints in other research focused only on stretching the MCP joint. In contrast, the device used in this study aimed to simultaneously stretch the MCP, PIP, and DIP joints. Based on the results of the ring and little fingers, the stretching effect applied to the MCP joints was not diluted but was, rather, superior to that of the devices used in previous research.

Similar to the other literature, the average pre–post difference obtained in this study (22.3 degrees) was 7.3 degrees higher than that obtained with surgical intervention [21], providing the participant with a superior therapeutic effect.

Therefore, the MCP joint achieved a functional ROM and approached a normal ROM. Although the improved ROM of directly traumatized little fingers was less than the functional ROM, the efficacy was still better than that observed in most previous studies.

#### 4.1.2. PIP

The flexion ROM of the PIP joints in the three fingers exceeded the functional angle [5,18] by 31.7 degrees on average. The ROM performance of the middle finger exceeded the normal ROM by 4 degrees. However, the ROM of the little finger and index finger was 10 degrees less than the normal ROM of 100 degrees.

The difference between pre- and post-treatment in this study was increased by 24 degrees compared with the results of conventional treatment in Jun et al.’s study [20]. Similar to the cases of the TAM and MCP joints, this can be attributed to differences in the device designs and measurement timing. Differences in the device designs may have been due to the distal palmar accessory, which fixed the palmar elastic line closer to the palm surface, increasing the flexion of the PIP joint.

The improvement was by 11.4 degrees compared with surgical intervention [21].

Therefore, the 7-week conventional treatment using the proposed device aided in restoring functional activity of the PIP joints, with the potential to return to the normal ROM.

#### 4.1.3. DIP

The flexion ROM of the PIP joints in the three fingers exceeded the functional [18] angle by 8 degrees on average but could not reach the normal ROM (70 degrees), with an average deficit of 23 degrees.

Compared with other conventional treatments [20], the average difference in the flexion ROM exceeded that obtained in previous research by 19.3 degrees. The improvement was 9.3 degrees greater compared with the literature on surgical intervention [21]. The greater degree of flexion was achieved due to the device design in this study, which included two factors for stretching the DIP joint: (1) the dorsal elastic line passing through the palmar surface of the fingertip, and (2) the distal palmar accessory tightening the palm-side elastic cord toward the palm surface. Although the pre–post difference for the ring finger (9 degrees) was slightly lower than in a previous surgical intervention study (12 degrees) [21], the ring finger exhibited a flexion ROM of 43 degrees in week 4, a difference of 13 degrees compared with pre-intervention. However, the angle was less than 43 degrees after the fourth week, indicating unavoidable measurement errors.

Although none of the fingers were able to regain a normal ROM, they all reached a functional ROM after 7 weeks of using the assistive device, which could be beneficial for performing hand activities.

### 4.2. TPD

The TPD in this study served as an efficient indicator of whether full flexion was reached. Macdermid et al. [23] showed that the TPD was related to the DASH score outcome, which represents a functional evaluation. In their study, the TPD values were 3.8 cm and 2.8 cm, while the DASH scores were above 20 and below 20, defined as substantial and minimal disability, respectively. The results in this study showed a similar tendency, with TPD values of 4.1 cm and 0.6 cm on average, while the DASH scores were 25.9 and 9.5 in week 0 and week 7, respectively.

In week 7, the TPD values of the middle and ring fingers were reduced to 0 cm, indicating the full flexion of the finger; however, a distance of 1.8 cm remained between the fingertip of the little finger and palm. The inconsistency between the injured fingers may be attributed to the initial states of the injured sites. The participant suffered fractures of the fifth metacarpal bone caused by falling. Considering this precondition, the little finger that was directly injured may have had a relatively worse prognosis.

### 4.3. DASH Score

In week 7, the DASH score was reduced to 9.5 points, which was within the normal range of healthy individuals aged 55–59 [24].

According to a previous study [25], a minimal clinically important difference (MCID) in the DASH score of at least 15 points qualified as an improvement in the function of the upper extremities. As such, our result of a 16.4-point difference was enough to prove the participant’s progress. This finding aligned with the increased TAM values of the three fingers compared with the functional ROM.

Many previous studies have researched the functional outcomes after distal radius fracture (DRF), ulnar styloid fracture, and metacarpal bone fracture [26,27,28]. To the best of our knowledge, whether DRF combined with metacarpal bone fracture influences the DASH score remains unknown. However, a combination with an ulnar styloid fracture would not have a significant impact [29]. We obtained lower DASH scores than the average DASH scores obtained in those studies by 18.8 points, indicating better functional outcomes. Nevertheless, we could not determine an exact explanation for this inconsistency because the previous studies did not record the design of the orthoses or the detailed rehabilitation procedures.

In Wang et al.’s study [22], the participants with MCP joint stiffness underwent a 5′8-week rehabilitation program using a static progressive splint. Their result of the improvement in the DASH score was 16.5 points, which was almost identical to ours. The orthosis-wearing timespan in their study was much longer than ours, lasting up to the whole night.

**Figure 11:**
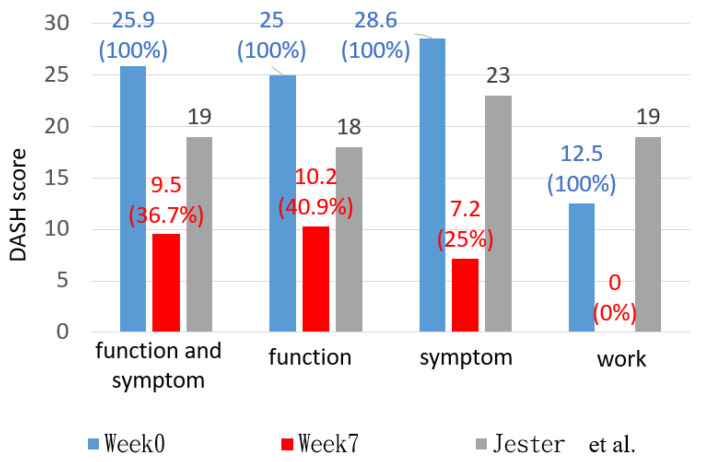
DASH score of each section in weeks 0 and 7 in comparison with the previous study [30].

The DASH score results were separately calculated in terms of function, symptoms, and work. These results were compared with the results of two workers aged between 50 and 65 years presented by Jester et al. [30]. In this study, the scores of the participant in weeks 0 and 7 were higher and lower than those obtained in previous studies, respectively (Figure 11). This indicates that the proposed device produced good rehabilitation for hand function. The results in week 7 regarding work showed no difficulties, indicating that participants were able to regain the necessary hand functionality for their occupations via the proposed device.

### 4.4. Features of the Developed Device

The new device exhibited some advantages compared with some other devices [13,14,15,16], as listed in Table 3. However, our device did not employ an external power assistant, and the patient needed to contract their muscles to perform the exercises [31]. The elastic lines utilized in this study were selected from commercially available resources. However, during the 7-week usage period, the palmar elastic line of the little finger and the dorsal elastic line of the ring finger each experienced one breakage, necessitating repair by the researcher. This raises some concerns regarding the durability of this type of elastic line. Furthermore, the process for determining the number of this particular elastic line used in the device was not standardized but according to the patient’s injury condition.

### 4.5. Limitation

This preliminary analysis recruited only one participant. While the results demonstrate that the device could increase the flexion ROM of the finger joints, decrease the TPD, and enhance the subjective functional outcome, the limited sample size could not generate sufficient evidence of its clinical efficacy.

The usage context for the device in this study was set as home-based rehabilitation exercises. Despite researchers providing pre-instructions for device usage, daily exercise plans, and regular reminders, the actual usage by the participant remained unknown, and the exercise amount was restricted by the participant’s work or schedule.

In this study, readings were visually obtained when conducting joint ROM measurements using goniometers. The readings might include errors. Only one researcher conducted the follow-up and outcome measurement, which may have exacerbated this bias; thus, interobserver reliability was not ensured in this study. Additionally, the length of the recovery period also depended on factors such as the patient’s initial injury severity, the operation type, the postoperative immobilization period, and the patient’s compliance with rehabilitation [32].

## 5. Conclusions

This study aimed to develop a dynamic finger flexion orthosis using AM technology, which could eliminate the drawbacks of traditional orthoses by effectively stretching each finger joint (especially the DIP joint) and allowing more hand functional activity to be performed. The results of this preliminary case study show that the 3DP-customized dynamic finger flexion orthosis enabled more functional activities and stretched all stiff joints in the finger. A 7-week home-based rehabilitation program for the orthosis increased the TAM by 53.6% on average. The developed 3DP orthosis was proven to improve hand function and produce a more complete ROM in finger flexion. The outcomes of this study could provide a theoretical reference for future relevant studies on this issue.

## Figures and Tables

**Table 1:** Demographic information.

Item	Detail
Age/sex	59/female
Diagnosis	Right distal radius fracture (DRF) Right ulnar styloid fracture Right fifth metacarpal bone fracture
Dominant hand	Right hand
Injury cause	Falling while walking
Injured finger	Middle finger, ring finger, and little finger

**Table 2:** Difference of DASH score over 7 weeks.

DASH Score	Sections I and II: Activity and Symptoms	Section I: Activity	Section II: Symptoms	Section III: Work
Week 0	25.9	25	28.6	12.5
Week 7	9.5	10.2	7.1	0
Difference	−16.4	−14.8	−21.5	−12.5

**Table 3:** Advantages and disadvantages of the developed device.

Advantages	Disadvantages
Cheap (material cost: USD 7) Light (150 g) Adjusts elastic line tension for the patient Finger reaches palm (maximum ROM) Offers functional training during the rehabilitation program Easy-to-fit joint location using a type of glove	No external power assistance The strength and durability of elastic lines need to be tested

## Data Availability

The data presented in this study are available upon request from the corresponding author.
